# Astrovirus as a possible cause of congenital tremor type AII in piglets?

**DOI:** 10.1186/s13028-014-0082-y

**Published:** 2014-12-16

**Authors:** Anne-Lie Blomström, Cecilia Ley, Magdalena Jacobson

**Affiliations:** Department of Biomedical Sciences and Veterinary Public Health, Faculty of Veterinary Medicine and Animal Science, Swedish University of Agricultural Sciences, SE-750 07 Uppsala, Sweden; Department of Clinical Sciences, Faculty of Veterinary Medicine and Animal Science, Swedish University of Agricultural Sciences, SE-750 07 Uppsala, Sweden

**Keywords:** Astrovirus, Circovirus, Congenital tremor, Myelin vacuolation

## Abstract

**Background:**

Congenital tremor is associated with demyelination of the brain and spinal cord and is clinically noted as outbreaks of trembling and shaking in newborn piglets during a limited time-period. Six forms of the disease have been described, where form AII may be caused by an, as yet, unidentified viral infection. This study aimed to investigate the presence of astrovirus and circovirus by sequencing and polymerase chain reaction (PCR) analysis and by relating the findings to the occurrence of disease and lesions in the brain, in 4–6 days-old piglets obtained from a clinical outbreak of congenital tremor.

**Results:**

In piglets with congenital tremor, there were mild to moderate vacuolar changes of the white matter in the cerebrum, brain stem and cerebellum. In healthy piglets, less conspicuous vacuolar changes were detected. One healthy and one diseased piglet were positive for porcine circovirus type 2. The nested pan-PCR showed the presence of astrovirus in at least one brain region in all piglets and by sequencing, two different porcine astrovirus lineages were identified.

**Conclusions:**

The results do not support previous studies identifying porcine circovirus type 2 as the cause of congenital tremor. The demonstration of astrovirus in the brain of piglets suffering from congenital tremor is interesting. However, astrovirus was demonstrated in both healthy and diseased individuals and therefore, further studies are warranted to determine the possible involvement of astrovirus in the pathogenesis of congenital tremor in pigs.

## Background

Congenital tremor (*myoclonia congenita)* in pigs was first described in 1922 [1]. Based on the lesions six forms of the disease, denoted AI-V and B, have been described: AI is related to transplacental infection with classical swine fever virus, AIII and AIV are judged as hereditary, AV is caused by a toxic substance [2] and form B is classified based on the absence of morphological lesions [3-5]. The form AII may be caused by an, as yet, unidentified viral infection [4,6] and has been reproduced by experimental inoculation of pregnant sows with tissues from affected piglets [2,7]. However, studies on the disease are hampered by its sporadic and occasional occurrence in the pig herds. In recent years, some studies have suggested a relationship to porcine circovirus type 2 (PCV2) [8,9], although this association is debated [10,11]. Microscopically, all forms of type A are associated with demyelination of the brain and spinal cord and in both infectious forms (AI and AII); a reduction in white and grey matter is described. No obvious deficiency of oligodendrocytes is noted but myelin deposition in existing sheaths seems to be retarded [7]. Cerebellar hypoplasia has only been noted in AI and AV [2,3]. Clinically, a few to all newborn piglets are trembling and shaking. The tremor is usually more severe in aroused piglets and generally abates with increasing age. In AI, the mortality is high but in AII, piglet mortality is usually low and mainly caused by malnutrition [4,12]. In other animal species, various causes of congenital tremor have been described. Recently, viral metagenomic studies revealed a relationship between “Shaking mink syndrome” and astrovirus (AstV) [13]. In pigs, a high number of animals have antibodies against AstV but the virus has so far only been related to mild diarrhoea in the presence of other enteric pathogens [14]. One study only describes the systemic infection with AstV [15].

In the present study, piglets from a herd suffering from a limited outbreak of congenital tremor were investigated. The aim was to study the presence of AstV and PCV2, and relate the findings to the occurrence of clinical signs of disease and lesions in the brain.

## Methods

### Herds and animals

The herd was an organic piglet-producing farm located approximately 250 km north of Uppsala, Sweden and keeping 160 sows in batch-wise production with 20 sows farrowing every third week. In October 2011, 10 recruitment gilts (Swedish Landrace × Yorkshire breed) were bought from a conventional gilt-producing herd that previously had experienced an outbreak of congenital tremor (P. Wallgren, pers. comm.). In the organic farm, five litters from 21 sows that farrowed in December 2011 suffered from congenital tremor. In the subsequent three batches, 31% of the litters included single piglets with congenital tremor (herd owner, pers. comm.). In March 2012, the outbreak had ceased. In total, six piglets were included in this study. Two piglets with distinct tremor but otherwise clinically healthy aged 4 days and one piglet aged 6 days from two of the first litters were selected. From the first batch of piglets born after the cessation of the outbreak, three clinically healthy, one-day-old piglets from two different litters were obtained and included as control pigs.

### Sampling

The sampling was approved by the Ethics Committee for Animal Experimentation, Uppsala, Sweden, and the herd owner had given an informed consent prior to the study.

The pigs were euthanized on the farm by intraperitoneal injection of pentobarbital-sodium (200 mg/mL, Pharmasol Ltd, Andover, UK). Immediately thereafter, the brain was aseptically removed from the skull and thin slices of the left and right cerebral hemispheres, the frontal lobe, and the cerebellum, were cut and frozen in liquid nitrogen. The remaining parts of the brain and, in the clinically healthy piglets, part of the spinal cord were placed in 10% buffered formalin. Specimens from other organs were not included.

### Pathology

Formalin-fixed material from brain and spinal cord was routinely embedded in paraffin. Limited amounts of brain tissue were available from the diseased piglets. Cerebral tissue was identified from all three piglets, part of brain stem in one piglet and cerebellum in one piglet. In healthy piglets most of the brain and spinal cord from all piglets were available for examination. Four-micrometer thick sections were mounted on microscope slides and stained with haematoxylin and eosin (H&E) and Luxol fast blue. Sections were examined by light microscopy using an Olympus BX41 microscope (Olympus America Inc., Melville, NY, USA).

### DNA extraction and PCV2 polymerase chain reaction

The DNA was extracted using the QIAamp DNA Mini Kit (Qiagen Inc., Hilden, Germany) according to the tissue protocol provided by the manufacturer and the DNA was eluted in 50 μL elution buffer (EB). All samples were screened for the presence of PCV2 using a SYBR-green polymerase chain reaction (PCR) targeting the in Sweden most commonly spread SG3 genotype as described by Blomström *et al.* [16].

### RNA extraction and cDNA synthesis

The extraction and PCR reactions were performed twice at separate occasions to control for the presence of false positive reactions.

The brain tissue was mechanically homogenized in 750 μL TRIzol (Invitrogen, Carlsbad, CA, USA). The RNA was extracted using a combination of TRIzol and RNeasy mini kit (Qiagen, Hilden, Germany) and the RNA was eluted in 30 μL EB. Two μL RNA was used in the cDNA synthesis step performed using Superscript III Reverse Transcriptase (Invitrogen) according to the manufacturer’s instructions using a mix of 150 ng random primers and 0.4 uM panAstV specific primer R1 (GGY TTK ACC CAC ATN CCR AA) [17].

### Nested PCR pan astrovirus (AstV)

The samples were screened for the presence of AstV by a nested pan-AstV PCR [17] designed to target a conserved region of the RNA-dependent-RNA polymerase (RdPp) gene that have previously been shown to efficiently identify AstV in humans [18], bats [17] and pigs [19,20]. In the first PCR, the primers panAV-F11, F12 and R1 was used and in the second PCR; the primers panAV-F21, F22 and R1. Each PCR was run under the following conditions: 1× PCR buffer, 2.5 mM MgCl_2_, 1.0 mM dNTP, 0.4 μM of each primer, and 1.25 U Ampli*Taq* Gold DNA polymerase (Applied Biosystems, Foster City, CA, USA). For each reaction, 2 μL cDNA or PCR-I product was used as template. The amplification was performed with the following reaction conditions: a 12 min enzyme activation step at 95°C followed by 39 cycles of 95°C for 30 sec, 58°C for 30 sec and 72°C for 90 sec, and finally, one cycle for 10 min at 72°C. The entire PCR II product was run on gel electrophoresis and the band of the expected size was cut, purified using the GeneJET PCR Purification Kit (Thermo Fisher Scientific Inc, Waltham, Massachusetts, USA) according to the manufacturer’s instructions, and eluted in 25 μL EB.

### Sequencing and analyses

The purified products were sequenced at Macrogen Europe (Amsterdam, the Netherlands) and the obtained chromatograms were edited using SeqMan (Lasergene 9, DNASTAR Inc., Madison, USA). ClustalW as well as the phylogenetic analysis were carried out using Mega 5 [21]. The phylogenetic trees were constructed using the Neighbour-joining algorithm with *P*-distances and with a bootstrap value of 1000. Sequence identity plots were performed using the BioEdit software (http://www.mbio.ncsu.edu/bioedit/bioedit.html). All the sequences from this study used in the phylogenetic analysis are deposited in Genbank (Accession numbers KC790414 to KC790418).

## Results

### Microscopic findings

**Figure 1:**
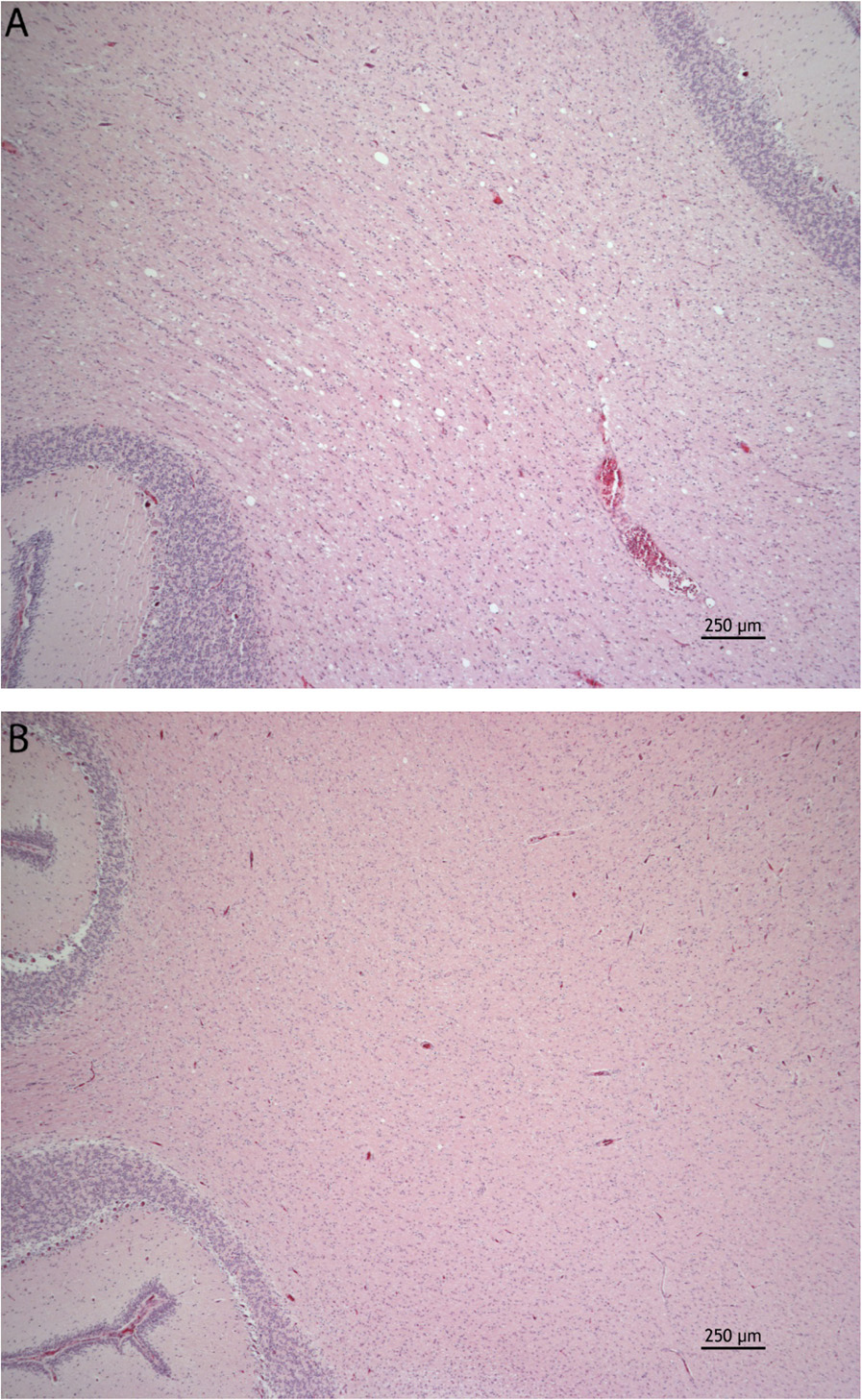
**Photomicrograph of an area of the cerebellum of A) a piglet affected by congenital tremor, showing numerous small vacuoles of the white matter and B) a healthy control piglet having minimal white matter vacuolation.** Haematoxylin and eosin.

**Figure 2:**
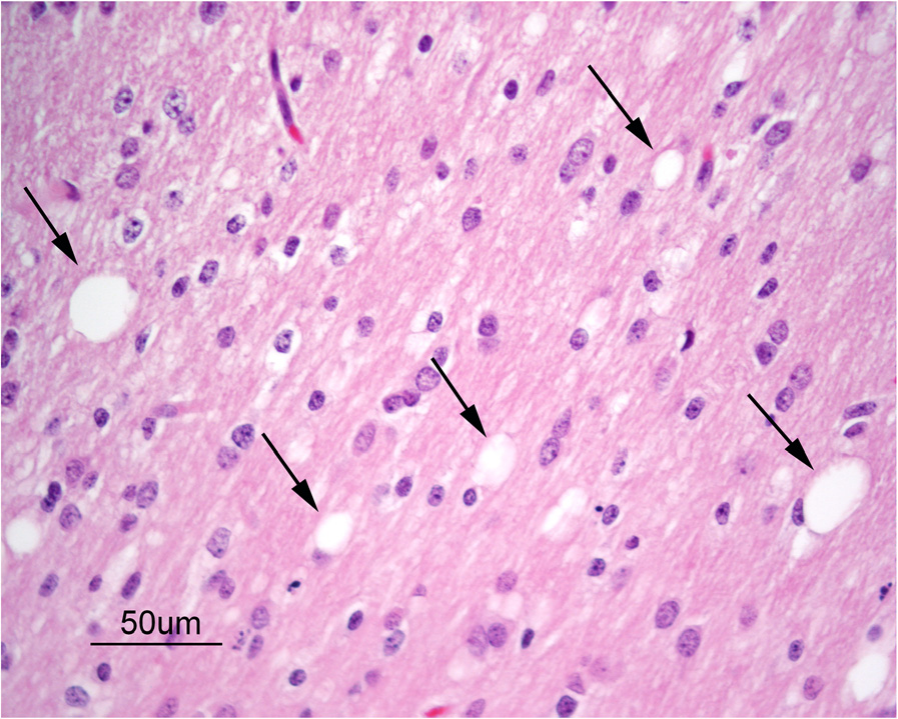
**Photomicrograph of cerebellar white matter of a diseased piglet showing vacuolar changes (arrows) at high magnification.** Haematoxylin and eosin.

In piglets with clinical signs of disease, there were mild to moderate vacuolar changes of the white matter in the cerebrum, brain stem and cerebellum. The most pronounced vacuolar changes were seen in the cerebellar white matter tracts (Figure 1A) with occasional vacuoles observed within the granular and molecular layers. Vacuoles typically presented as round to oval clear spaces of varying size with larger vacuoles measuring up to approximately 100 μm in diameter. Vacuoles appeared randomly scattered and were usually single, however sometimes they were seen coalescing or grouped together in small numbers (Figure 2). Also in healthy piglets slight vacuolar changes were detected, however these changes were less conspicuous than the vacuolar changes in diseased piglets (Figure 1B). Extravasated erythrocytes were variably present in the meninges and multifocally in neural tissue of both diseased and healthy pigs. Both in diseased and control animals cerebral sections often showed sparse Luxol fast blue staining for myelin, whereas brain stem and spinal cord sections of controls tended to be more intense. Staining for myelin in the cerebellum appeared similar in the section from the diseased pig and one control pig, but weaker than in the two other control animals (results not shown).

### PCV2 polymerase chain reaction

In the screening of PCV2, two samples were identified as positive – one healthy and one diseased piglet.

### Nested PCR pan astrovirus

The results from the nested pan-PCR showed the presence of AstV. Three of the samples (two control pigs and one diseased pig) were clearly positive whereas a number of the remaining samples had faint bands of the correct size. The extraction and PCRs were performed twice at separate occasions and although the intensity of the band varied, a positive PCR signal was demonstrated in at least one brain region in all piglets at both occasions.

### Sequence analyses

**Figure 3:**
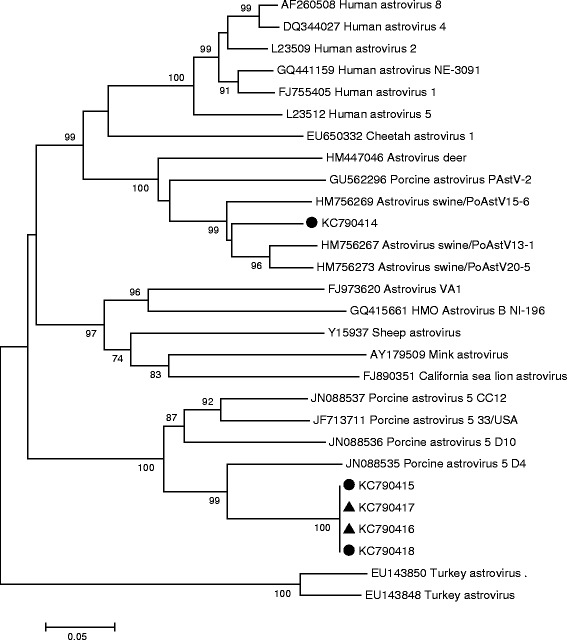
**Phylogenetic analysis of astroviruses demonstrated in brain tissue from piglets with congenital tremor (●) and in brain tissue from healthy control piglets (▲).** The phylogenetic analysis was performed using Neighbour-joining algorithm and bootstrap value of 1000. Only branch support over 70% is displayed in the figure. If several regions of the brain of one individual were positive for the same PoAstV linage, only one of the sequences was used in the tree.

To verify the results of the PCR, the three strong bands were sequenced as were a number of the faint bands. Sequence and BLAST analysis of the PCR products showed the presence of two different porcine AstV (PoAstV) lineages – PoAstV 5 [19], and PoAstV 2 [20]. This was also supported in the phylogenetic analysis (Figure 3). The sequences grouping with PoAstV 5 showed a 75.8 – 80% sequence identity to previously described PoAstV 5. Similarly, the PoAstV 2 from this study had a sequence identity of 90.1% to a previously described PoAstV 2 (PoAstV20-5). In the present study, sequences belonging to the same PoAstV group showed a 100% similarity to each other, whereas the similarity between the groups was 56.8% on nucleotide level. Both lineages were found in pigs with congenital tremor and in the control pigs and it was not possible to relate the findings to clinical disease.

## Discussion

The study presented here is based on a field outbreak of congenital tremor in an organic, piglet-producing herd. Our investigation shows the presence of two different porcine astroviral lineages, PoAstV 2 and 5, in the brain of newborn piglets. Interestingly, these lineages have, to our knowledge, not been reported in Swedish pigs before. Recent investigations have displayed a high genetic diversity among PoAstV with at least five different lineages circulating, and therefore a broad pan-PCR was used to screen the samples. The same PCR has previously been used in the first identification and characterisation of the PoAstV 2 in swine in Quebec, Canada [20] and the sequences from our study displayed a 90.1% identity to one of the PoAstV 2 (PoAstV20-5) sequences from that study. This PoAstV linage has also been identified in pigs in USA, China and South Korea. The PoAstV5 virus, discovered from this study, showed a 75.8 – 80% nucleotide sequence identity to the recently discovered PoAstV5 [19]. Astroviruses have been identified in a large number of both mammalian and avian species such as humans, felines, minks, bats and chickens [22] and they have been known to infect and replicate in pigs since the 1980s [23]. The majority of investigations concerning PoAstV relate to enteric disorders. However, in two metagenomic studies, AstV have been detected in association with neurological disorders [13,24]. In one of these studies on mink kits suffering from “Shaking mink syndrome”, mink-AstV was detected in both naturally diseased kits and in the brain from experimentally infected kits [13]. In the present study it was, however, neither possible to conclusively associate the presence of the specific PoAstV lineages nor the presence of PCV2 to clinical disease. It would have been desirable to include more piglets, but because of the short duration and sporadic occurrence of the clinical outbreak, combined with the farmers’ reluctance to sacrifice piglets for research purposes, this was not possible. Hence, the significance of our findings should be further investigated.

The lesions found in the present study are consistent with previous descriptions on pigs suffering from congenital tremor [7]. The vacuolation detected in the white matter in diseased piglets may relate to a hypomyelinogenesis, or a mild form of spongioform myelinopathy as seen in metabolic, toxic or idiopathic conditions. Myelin vacuolation is not an uncommon *post-mortem* artifact [25,26]. However, autolytic vesiculation of myelin or decrease in cholesterol content will probably not be noted during the first four hours post-mortem [22,27-29]. Luxol fast blue staining was used to evaluate the presence of myelin. However, the exact anatomic location of sampling sites was not known due to the slicing of the fresh brain, and hence it was not possible to fully evaluate the staining results in detail. Thus, the results from Luxol fast blue staining were considered uncertain. The observed extravasated erythrocytes likely represent *post-mortem* artifacts associated with the sampling procedure. At the start of the outbreak, the aim was to enable the rejection or support by PCR of the, at the time, rather speculative hypothesis and therefore, brain tissue was collected in accordance with a previously used protocol [13]. Future development of antibodies or probes to visualise virus in neural tissue, *e.g.* by protein detection using immunohistochemistry or by detecting viral genome by *in situ* hybridization, would highly strengthen the results and provide stronger evidence for AstV as a cause of congenital tremor in the diseased pigs.

The epidemiological pattern noted during this outbreak seems consistent with previous reports. Following introduction of the infection into a herd, congenital tremor may occur in most litters born over a period of two to three months with a high proportion of piglets affected in each litter [4]. In the present study, approximately 30% of the litters born during three months time were affected. In the first batch of piglets born following the cessation of the outbreak, the sows had been introduced to the recruitment gilts before mating. Hence, it is possible that they had been in contact with the virus and subsequently developed protective immunity before gestation.

## Conclusions

The demonstration of astrovirus in the brain of piglets suffering from congenital tremor is an interesting finding. However, our results do not conclusively identify neither AstV nor PCV2 as the cause of the disease as these viruses were found in both healthy and diseased individuals. Thus, further studies are warranted to elucidate the cause, using techniques such as broad PCR, pan microarrays and next generation sequencing technologies, together with experimental inoculation studies.
